# IgG4-related disease of duodenal obstruction due to multiple ulcers in a 12-year-old girl

**DOI:** 10.1186/s12887-023-04190-z

**Published:** 2023-07-25

**Authors:** Daiki Kato, Hiroo Uchida, Akinari Hinoki, Wataru Sumida, Chiyoe Shirota, Satoshi Makita, Masamune Okamoto, Aitaro Takimoto, Shunya Takada, Yoichi Nakagawa

**Affiliations:** 1 Department of Pediatric Surgery, Nagoya University Graduate School of Medicine, 65 Tsurumai-cho, Showa- ku, Nagoya, 466-8550 Japan; 2 Department of Rare/Intractable Cancer Analysis Research, Nagoya University Graduate School of Medicine, 65 Tsurumai-cho, Showa-ku, Nagoya, 466-8550 Japan

**Keywords:** IgG4-related disease, Gastrointestinal ulceration, Gastric outlet obstruction

## Abstract

**Background:**

Immunoglobulin G4-related disease (IgG4-RD) is a systemic inflammatory disease and affected individuals typically present with an increased infiltration of IgG4-positive plasma cells in the pancreas, hepatobiliary tract, and liver but rarely in the gastrointestinal tract.

**Case presentation:**

A 12-year-old girl presented with vomiting and poor weight gain. Gastroscopy revealed duodenal stenosis and ulceration. Computed tomography revealed edematous duodenal wall thickening and air-fluid levels on the right side of the duodenum, which suggested duodenal perforation or penetration. She underwent pancreaticoduodenectomy, and IgG4-RD was diagnosed via histopathology.

**Conclusions:**

This is the first pediatric case of isolated duodenal IgG4-RD resulting in duodenal obstruction after multiple ulcers. Gastrointestinal IgG4-RD should be among the differential diagnoses of unexplained gastrointestinal obstruction or ulceration even in children.

## Background

Immunoglobulin G4-related disease (IgG4-RD) is a systemic inflammatory disease which is characterized by an abundance of IgG4-positive plasma cell infiltration in the affected organs and an elevation in the serum IgG4 levels [1]. This condition is typically misdiagnosed as malignancy and patients respond well to steroid administration; furthermore, IgG4-RD is predominantly observed in elderly men [1, 2]. It typically manifests in the pancreas, hepatobiliary tract, and liver, it is rarely observed in the gastrointestinal tract [3]. There are a few cases of isolated gastrointestinal IgG4-RD have been reported, and there is only one case of a 59-year-old woman with duodenal obstruction [3]. We present the case of a 12-year-old girl with gastrointestinal IgG4-RD that resulted in duodenal obstruction after multiple ulcers and required pancreaticoduodenectomy. Furthermore, we have reviewed the relevant literature, emphasizing the diagnosis and treatment of IgG4-RD in a gastrointestinal region.

## Case presentation

**Fig. 1:**
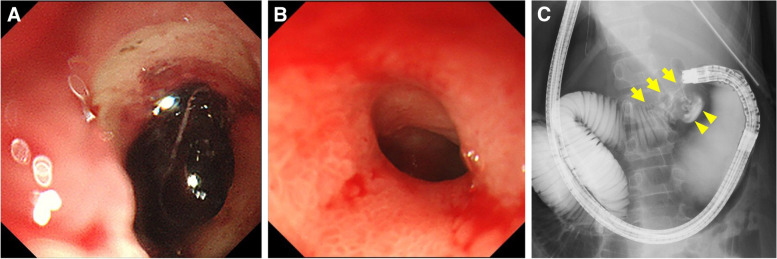
**A** Initial gastroscopy showing a duodenal ulcer and the adhesion of blood clots. **B** Second gastroscopy showing duodenal stenosis. **C** Duodenography showing duodenal stenosis (arrow) and tasche formation (arrow head)

The patient was a 12-year-old girl with no comorbidities and no oral medications. She fainted after hematemesis and hematochezia and was rushed to the ER. She received a blood transfusion for severe anemia and underwent gastrointestinal endoscopy, which detected a hemorrhagic duodenal ulcer (Fig. 1A). She was treated with a proton pump inhibitor (PPI) and her symptoms improved. One month later, she presented with vomiting and poor weight gain. She underwent gastroscopy, which revealed duodenal stenosis and multiple duodenal ulcers (Fig. 1B). Duodenography showed duodenal stenosis and tasche formation (Fig. 1C). Even a narrow endoscope (7.7 mm) could not pass through the stenosis, and a biopsy of the duodenal stricture was impossible. She was managed via total parenteral nutrition and referred to our hospital.

**Fig. 2:**
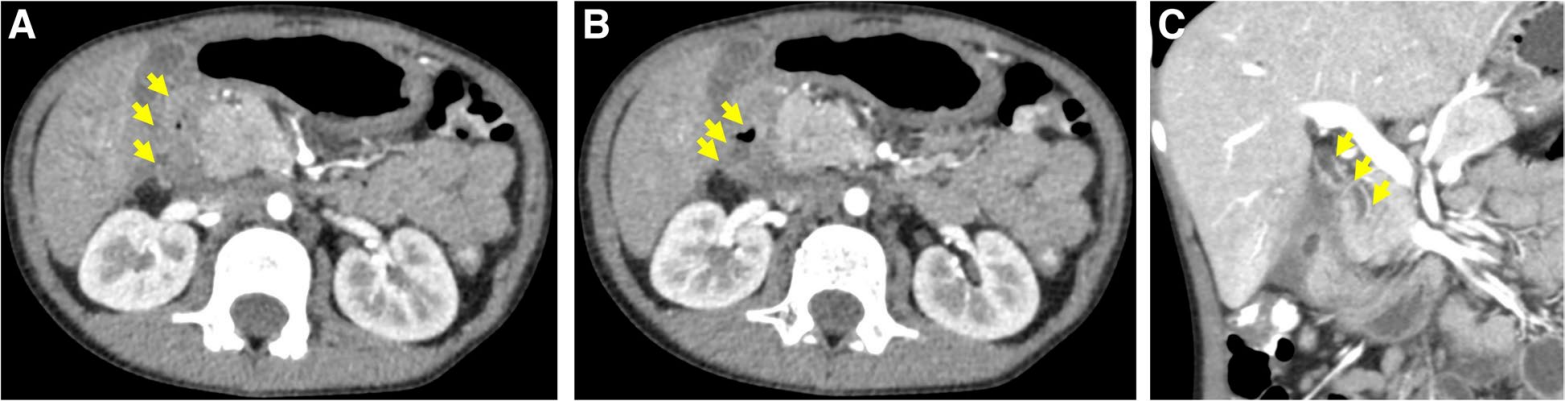
**A** Abdominal CT showing edematous duodenal wall thickening. **B** CT showing air-fluid levels on the right side of the duodenum. **C** The wall of the common bile duct is thickened with enhancement

Edematous duodenal wall thickening and air-fluid levels on the right side of the duodenum were observed via abdominal computed tomography (CT), which suggested the presence of duodenal perforation or penetration. The wall of the common bile duct was thickened with enhancement. There was no swelling or mass in the pancreas (Fig. 2A–C).

She had high serum levels of IgG4 (214 mg/dl) but no other tumor marker level was remarkable (CEA: 0.7 ng/ml, CA19-9: 7 U/ml). Serum helicobacter pylori antibody was negative. Serum gastrin was normal (87 pg/ml). C-reactive protein (0.19 mg/dl), total bilirubin (0.3 mg/dl), and amylase (115 U/l) were not elevated (Table 1).

**Table 1:** Blood test results

Item	Value	Item	Value
Asparate aminotransferase	30 U/l	c-reactive protein	0.19 mg/dl
Alanine aminotransferase	9 U/l	White blood cells	4700 /µl
Total bilirubin	0.3 mg/dl	hemoglobin	8.5 g/dl
γ-glutamyl transpeptidase	16 U/l	platelet	42.3 × 10^4^ /µl
Urea nitrogen	7.3 mg/dl	CEA	0.7 ng/ml
Creatinine	0.35 mg/dl	CA19-9	7 U/ml
Amylase	115 U/l	Helicobacter pylori antibody	negative
Glucose	126 mg/dl	gastrin	87 pg/ml
Sodium	140 mEq/l	Immunoglobulin G4	214 mg/dl
Potassium	4.2 mEq/l		
Chloride	106 mEq/l		

Two months of PPI treatment did not improve the obstruction and multiple duodenal ulcers. CT revealed suspected duodenal perforation or penetration. Hyper-inflammation of the duodenum could lead to the Vater’s papillary deformity and, eventually, obstructive jaundice and liver damage. A pathological diagnosis was not obtained preoperatively, thus malignancy could not be ruled out. Moreover, she was unable to feed for long and, consequently, experienced poor weight gain. Therefore, she underwent pancreaticoduodenectomy.

Gross examinations of the resected specimen revealed perforation and an ulcerated duodenal wall.

**Fig. 3:**
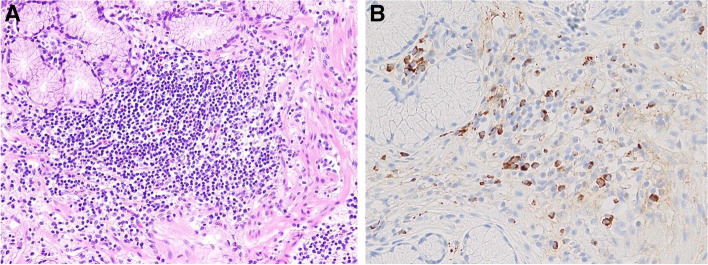
**A** There is extensive sclerosing fibrosis and lymphoplasmacytic inflammation (Hematoxylin-eosin stain, 200×). **B** Immunohistochemical staining revealed an increased number of IgG4-positive plasma cells (IgG4 stain, 200×)

Microscopically, a duodenal ulcer was observed, which was associated with the underlying presence of extensive sclerosing fibrosis, and the presence of lymphoplasmacytic inflammation which involved the duodenal wall (Fig. 3A). There was no malignancy. Immunohistochemical staining revealed numerous IgG4-positive plasma cells at 50/HPF, and the IgG4/IgG ratio was > 40% (Fig. 3B). There were no IgG4-positive cells in the bile duct or pancreas.

Following an uneventful postoperative course, the patient was discharged on the postoperative day 16 tolerating oral intake. The serum IgG4 level decreased to 117 mg/dl postoperatively. She is currently doing well one month after surgery.

## Discussion and conclusions

IgG4-RD is a rare inflammatory condition of the gastrointestinal tract that may lead to gastric ulceration; this complication typically occurs following the detection of fibrosis along with lymphoplasmacytic infiltrates and lymphoid follicles within the ulcer [3]. Numerous prior reports primarily aimed to examine pancreatic involvement, and this condition is almost interchangeably called autoimmune pancreatitis [1].

Our patient presented with duodenal obstruction, a rare complication of IgG4-RD. To the best of our knowledge, there were 11 reported cases in the literature to date with gastrointestinal luminal obstruction. The patients’ ages were > 20 years in all reported cases. There were two cases of gastric outlet obstruction; one was secondary to pancreatitis [4] and the other was isolated duodenal IgG4-RD [3]. Therefore, ours is possibly the first reported pediatric case of isolated duodenal IgG4-RD resulting in gastric outlet obstruction. Endoscopic ultrasound-guided fine-needle aspiration yielded the diagnosis in two cases [4, 5]. Similar to our case, most of reported cases required surgery to reach the diagnosis.

The IgG4-RD diagnostic criteria are as follows: (1) characteristic diffuse/localized swelling or masses in single or multiple organs upon clinical examination, (2) a serum IgG4 concentration of ≥ 135 mg/dl, and (3) a remarkable degree of lymphocytic and plasma cell infiltration along with fibrosis and the infiltration of IgG4-positive plasma cells, with a ratio of IgG4/IgG-positive cells of > 40% and > 10 IgG4-positive plasma cells/HPF on histopathological examinations [6].

Steroid therapy can be considered a viable treatment strategy when the diagnosis of gastrointestinal IgG4-RD is achieved without surgical resection. If a definitive diagnosis was obtained by examinations such as core needle biopsy or laparoscopic exploration and biopsy, we could try the steroid therapy. High-risk surgical resections such as pancreaticoduodenectomy would have been performed only when steroid therapy was ineffective. However, isolated gastrointestinal IgG4-RD is particularly difficult diagnose before surgical resection owing to the fact that it is a considerably rare condition and presents with features similar to malignancy. IgG4-RD is diagnosed via histopathology with IgG4 staining [6]. As described above, surgical resection should be the final resort to reach the diagnosis. In fact, we opted for surgery after consultation with gastroenterologist because there are few reports of this disease in the literature.

Gastrointestinal IgG4-RD should be among the differential diagnoses of unexplained gastrointestinal obstruction or ulceration even in children. If there is a situation in which IgG4-RD is suspected, a biopsy should be performed to make the diagnosis whenever possible, especially in children. Once IgG4-RD is definitively diagnosed, medical treatment including steroids should be administered, and surgical resection should be performed only if the disease is refractory to medical therapy.

## Data Availability

The data presented in this study are available on reasonable request from the corresponding author.

## Abbreviation

IgG4-RD: Immunoglobulin G4-related disease
